# Pharmacokinetics and Tissue Distribution of Bee Venom-Derived Phospholipase A2 Using a Sandwich ELISA after Subcutaneous Injection of New Composition Bee Venom in Rats

**DOI:** 10.3390/ijms241210214

**Published:** 2023-06-16

**Authors:** Soon Uk Chae, Seong Jun Jo, Chae Bin Lee, Sangyoung Lee, Ji-Hyun Park, Jin-Su Jung, Eui-Suk Park, Hyunsu Bae, Soo Kyung Bae

**Affiliations:** 1 College of Pharmacy and Integrated Research Institute of Pharmaceutical Sciences, The Catholic University of Korea, 43 Jibong-ro, Bucheon-si 14662, Republic of Korea; zldtnseo@catholic.ac.kr (S.U.C.); seongjun6734@catholic.ac.kr (S.J.J.); aribri727@catholic.ac.kr (C.B.L.); nssy0416@catholic.ac.kr (S.L.); 2 INISTst R&D Center, 19th F, Higgs U-Tower, 184, Jungbu-daero, Yongin-si 17095, Republic of Korea; parkjh@inistst.com (J.-H.P.); js@inistst.com (J.-S.J.); espark@inistst.com (E.-S.P.); 3 Department of Physiology, College of Korean Medicine, Kyung Hee University, Seoul 02453, Republic of Korea; hbae@khu.ac.kr

**Keywords:** new composition bee venom, bee venom-derived phospholipase A2, pharmacokinetics, tissue distribution, rats

## Abstract

Bee venom is a traditional drug used to treat the nervous system, musculoskeletal system, and autoimmune diseases. A previous study found that bee venom and one of its components, phospholipase A2, can protect the brain by suppressing neuroinflammation and can also be used to treat Alzheimer’s disease. Thus, new composition bee venom (NCBV), which has an increased phospholipase A2 content of up to 76.2%, was developed as a treatment agent for Alzheimer’s disease by INISTst (Republic of Korea). The aim of this study was to characterize the pharmacokinetic profiles of phospholipase A2 contained in NCBV in rats. Single subcutaneous administration of NCBV at doses ranging from 0.2 mg/kg to 5 mg/kg was conducted, and pharmacokinetic parameters of bee venom-derived phospholipase A2 (bvPLA2) increased in a dose-dependent manner. Additionally, no accumulation was observed following multiple dosings (0.5 mg/kg/week), and other constituents of NCBV did not affect the pharmacokinetic profile of bvPLA2. After subcutaneous injection of NCBV, the tissue-to-plasma ratios of bvPLA2 for the tested nine tissues were all <1.0, indicating a limited distribution of the bvPLA2 within the tissues. The findings of this study may help understand the pharmacokinetic characteristics of bvPLA2 and provide useful information for the clinical application of NCBV.

## 1. Introduction

Bee venom has been used in traditional medicine for thousands of years for various diseases, such as those of the nervous system, musculoskeletal system, and autoimmune disorders [1]. Bee venom, as a toxin produced by honeybees (*Apis mellifera*), consists of various peptides, including melittin (50–60%), apamin (1–3%), adolapin (0.1–0.8%), and mast cell degranulating peptide (1–3%). It also contains enzymes such as phospholipase A2 (PLA2; 10–12%), biologically active amines (e.g., histamine and epinephrine), and non-peptide components (e.g., minerals and amino acids). The major components of bee venom are known to have various biological activities, such as anti-cancer, anti-inflammatory, and neuronal activation [2,3,4]. Apitox^®^, majorly composed of melittin, was approved for the treatment of patients with osteoarthritis by the US FDA following a phase 3 clinical study and is currently used for patients with arthritis in the Republic of Korea [5,6].

**Figure 1:**
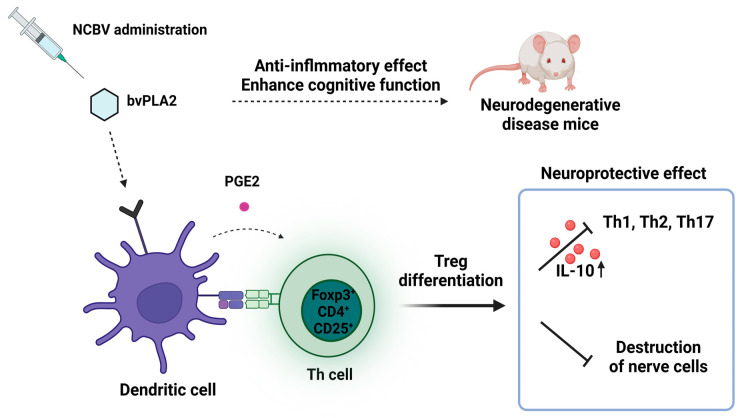
Potential therapeutic mechanism of bvPLA2 (group III secretory PLA2) for neurodegenerative diseases such as Alzheimer’s disease.

**Figure 2:**
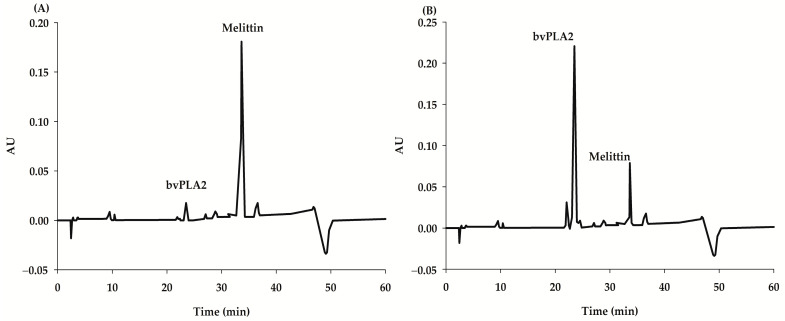
HPLC chromatograms showing the content of bvPLA2 (group III sPLA2) in crude bee venom (**A**) and NCBV (**B**); calculated as 76.2% bvPLA2).

Among the various components contained in bee venom, bee venom-derived PLA2 (bvPLA2; GenBank accession no. AFI40558.1), which belongs to group III secretory PLA2 with a molecular weight of 14 to 18 kDa, is known to have various pharmacological effects, including an anti-neurodegenerative effect, an anti-inflammatory effect, an anti-nociceptive effect, an anti-cancer effect, and an anti-bacterial effect [7]. Through a non-clinical test, bvPLA2 (group III sPLA2) has been shown to activate Tregs and promote the production of IL-10, which helps inhibit the differentiation of helper T1, T2, and Th17 cells and destruction of nerve cells by microglia (Figure 1); these processes were confirmed to be neurodegenerative diseases such as Parkinson’s disease and Alzheimer’s disease accompanied by inflammatory reactions [8,9,10]. In an animal model of neurodegenerative disease, bvPLA2 not only showed neuroprotective and anti-inflammatory activity but also improved memory-related function [9,11]. Previous research has shown that bvPLA2 (0.2 and 1 mg/kg) enhances cognitive function by increasing the Treg population, inactivating microglia, and reducing CD4+T cell infiltration in the triple-transgenic Alzheimer’s disease mouse model [12]. Additionally, it was reported that bvPLA2 (1 mg/kg) improved memory deficiency and cognitive impairment in Tg2576 mice, which are widely used as another Alzheimer’s disease mouse model, via inhibiting the STAT3 signaling pathway [13]. These results provide evidence that bee venom and bvPLA2, one of its components, may protect the brain from neurological disorders and have potential for use in the treatment of Alzheimer’s disease. Although the dried crude honeybees usually consist only of approximately 10–12% of bvPLA2 (Figure 2A) [3,4], based on the proven efficacy of bee venom and bvPLA2 in Alzheimer’s disease, a new composition of bee venom (NCBV; Figure 2B) was developed with a fortified content of bvPLA2 (up to 76.2%). From crude honeybee, NCBV was lyophilized using an ultrafiltration manufacturing process by the INISTst R&D Center (Gyeonggi-do, Republic of Korea) [14]. 

As mentioned earlier, the NCBV is composed of complex mixtures such as peptides, phospholipids, bioactive amines, amino acids, sugars, pheromones, enzymes, and minerals. The main component of NCBV is bvPLA2, a peptide with a molecular weight of 14 to 18 kDa that is also composed of a complex mixture. Currently, most peptide drugs are administered by the parental route, such as intravenous, subcutaneous, and intramuscular injections [15]. These routes have the advantages of avoiding pre-systemic metabolism from hepatic and gastrointestinal first-pass effects and achieving complete systemic availability of the drug at the target site. Intraperitoneal injection of bvPLA2 or NCBV in Alzheimer’s disease mouse models has been shown to improve memory-related functions as well as neuroprotective and anti-inflammatory actions [8,9,10,11,12,13]. However, intraperitoneal injection is minimally used in clinical practice. In our preliminary study, subcutaneous injections in rats confirmed that NCBV can penetrate the skin and interact with immune cells, nerve endings, and other targets in local tissues. Considering (1) that NCBV is composed of multiple components, (2) that the main component, bvPLA2, is a peptide with a molecular weight of 14–18 kDa, and (3) our preliminary studies following subcutaneous injection, in this study, we have evaluated pharmacokinetic studies for bvPLA2 in rats through subcutaneous injection of NCBV. Therefore, we are also planning a phase I clinical trial to administer NCBV subcutaneously to patients with Alzheimer′s disease in the Republic of Korea (INISTst R&D Center).

Prior to clinical studies, a comprehensive understanding of non-clinical in vitro and in vivo pharmacokinetics is essential to establishing the efficacy, toxicity, and safety profiles of new investigational drugs. In this study, we evaluated the pharmacokinetic profile of bvPLA2 after subcutaneous (SC) or intravenous (IV) administration of various doses of NCBV in rats for the first time. In addition, a comparative study of bvPLA2 pharmacokinetics following NCBV and pure bvPLA2 administration at equivalent doses was performed to investigate whether other constituents of NCBV have an effect on the pharmacokinetics of bvPLA2. To the best of our knowledge, no analytical method for measuring bvPLA2 in biological samples has been reported to date; thus, we developed a sandwich ELISA method for the quantification of bvPLA2 in biological samples.

## 2. Results and Discussion

### 2.1. Minimum Required Dilution for ELISA Assay

The minimum required dilution was determined using two replicates of blank rat plasma spiked with bvPLA2. Plasma samples were diluted at 1:2, 1:4, 1:10, and 1:20 in the dilution buffer to a final concentration of 25 ng/mL (*n* = 5 for each dilution). For each dilution, the accuracy ranged from 96.4% to 106% (Table 1). This result suggests that there was no interference in the blank plasma and a 1:2 dilution in the buffer. Thus, the samples could be diluted up to 20-fold. All samples used in the assay were diluted to at least 1:2 to avoid nonspecific matrix interference.

### 2.2. ELISA Assay Validation

The intra-day precision and accuracy were used to evaluate the efficiency of the sandwich ELISA method by measuring different concentrations of bvPLA2 quality control (QC) samples (100, 80, 10, 2, and 0.78 ng/mL). Inter-day precision and accuracy assessments were based on the measurement of bvPLA2 levels for six independent batches. The results indicate that the sandwich ELISA was successfully applied to measure bvPLA2 in the biological samples of plasma and tissues. The range of the calibration curve for plasma and various tissues was 0.78–100 ng/mL, and all samples had an *S*-curve with a correlation coefficient (R^2^) greater than 0.998. The lower limit of quantification (LLOQ) was 0.78 ng/mL. The precision of QC samples for plasma and all tissues was within the range of 102% to 111% and 109% for intra-day and inter-day, respectively. The accuracy of QC samples for plasma and all tissues was within the range of 96.7% to 113% and 114% for intra-day and inter-day, respectively. Our developed ELISA method was performed according to the US FDA bioanalytical method validation guidance for industry [16]. Therefore, this sandwich ELISA could be applied to the determination of bvPLA2 in biological samples. 

### 2.3. Pharmacokinetic Studies of bvPLA2 after Single Intravenous Administration of NCBV in Rats at a Dose of 0.05 mg/kg 

**Figure 3:**
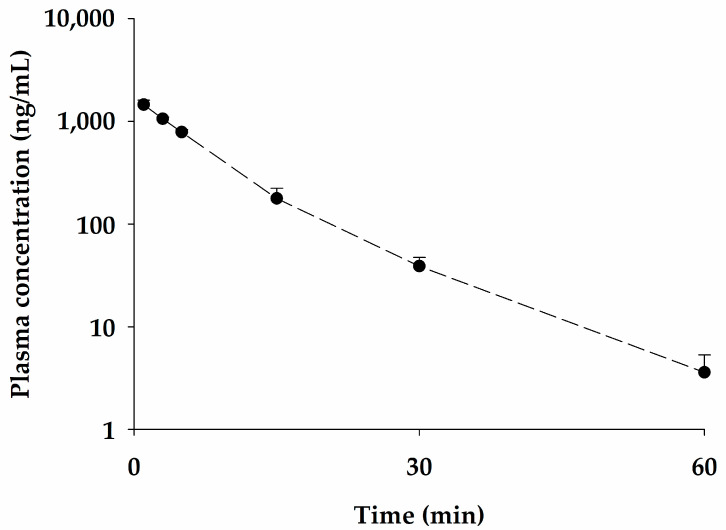
Mean plasma concentration-time profile for bvPLA2 following intravenous administration of NCBV at a dose of 0.05 mg/kg (●). Vertical bars represent SD.

The newly developed ELISA was used in the pharmacokinetic study of bvPLA2 in rats after IV administration of NCBV at a dose of 0.05 mg/kg. The plasma concentration-time profile of bvPLA2 in rats is shown in Figure 3, and the relevant pharmacokinetic parameters are listed in Table 2. As shown in Figure 3, bvPLA2 was detected in rat plasma for up to 120 min after administration. After IV administration, the plasma concentration of bvPLA2 rapidly decreased, with an elimination half-life (t_1/2_) of 8.10 ± 0.669 min and a total body clearance (CL) value of 4.24 ± 0.291 mL/min/kg. The apparent volume of distribution at steady state (V_dss_) was considerably small at 49.3 ± 1.79 mL/kg, slightly larger than a plasma volume (~40 mL/kg), indicating very limited distribution within the tissues of the bvPLA2 and largely confined to the plasma space. The V_dss_ of therapeutic peptides is generally small and limited to the extracellular space [17]. The mean residence time (MRT) was also short at 8.11 ± 1.07 min, suggesting that bvPLA2 was rapidly eliminated from the body and does not remain in circulation for an extended period. bvPLA2 was not detectable in the urine over 24 h (Ae_24 h_), suggesting that the bvPLA2 was not distributed to the kidneys for elimination or that it had been metabolized or degraded and eliminated from the body before it could be excreted in the urine. 

### 2.4. Pharmacokinetic Properties of bvPLA2 after a Single Subcutaneous Administration of NCBV in Rats at Various Doses 

**Figure 4:**
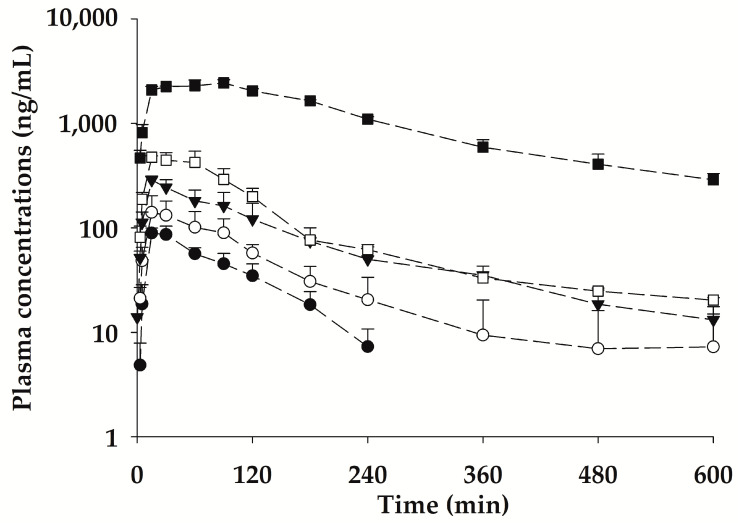
Mean plasma concentration-time profiles for bvPLA2 after subcutaneous administration of NCBV (0.2 mg/kg: ●, 0.5 mg/kg: ○, 1 mg/kg: ▼, 2 mg/kg: □, and 5 mg/kg: ■). Vertical bars represent SD.

**Figure 5:**
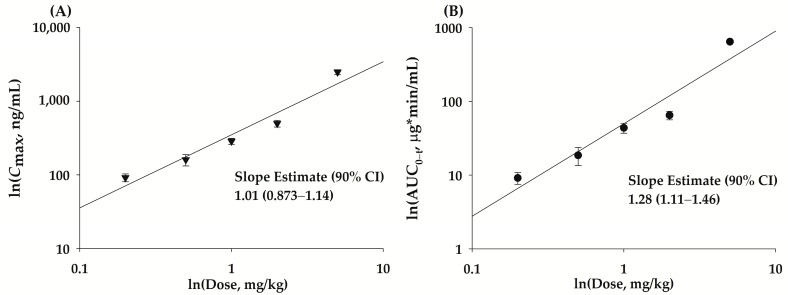
Assessment of dose proportionality for bvPLA2 *C*_max_ (**A**, **▼**) and AUC_0−t_ (**B**, **●**) following single SC administration of NCBV ranging from 0.2 to 5 mg/kg. Vertical bars represent SD.

The mean plasma concentration-time profiles of bvPLA2 after SC administration of NCBV at doses of 0.2, 0.5, 1, 2, and 5 mg/kg in rats are shown in Figure 4, and the relevant pharmacokinetic parameters are listed in Table 3. After SC administration of NCBV, bvPLA2 was detected in the plasma at the first blood sampling time point (3 min) and reached *C*_max_ rapidly (15–60 min; Table 3) in all dose groups. A short *T*_max_ for bvPLA2 suggests that the absorption of bvPLA2 from the rat subcutaneous tissue (hypodermis) was fast and its systemic circulation was rapid. The mean plasma concentration increased in a dose-dependent manner but was not detected in any group at the last sampling point (1440 min). The *C*_max_ and AUC_0−t_ values also showed a dose-dependent increase; in particular, the dose proportionality for *C*_max_ was confirmed by the 90% confidence intervals (CIs) for slopes of log transformed dose, and *C*_max_ was outside the limits of 0.93–1.06. Furthermore, it was concluded that AUC_0−t_ was not proportional because the 90% CIs for slopes of log transformed dose and AUC_0−t_ were outside the limits of 0.93–1.06 (Figure 5, Table 4). The AUC_0−t_ value in the highest dose group (5 mg/kg) may have led to non-proportionality of bvPLA2, as the dose-normalized AUC_0−t_ value was higher than that of the other dose groups. The saturable binding of bvPLA2 to plasma proteins or its distribution to tissues may be attributable to the observed nonlinearity of the AUC_0−t_ and *C*_max_. The considerably decreased apparent distribution volume (V_dss_/*F*) at the highest dose of 5 mg/kg (1.34 L/kg) compared to other dose groups (4.49–6.95 L/kg) provides evidence that bvPLA2 was highly exposed to systemic circulation (Table 3). At the lowest dose of NCBV (0.2 mg/kg), the V_dss_/*F* could not be compared with that of 5 mg/kg because the plasma levels of bvPLA2 were obtained only up to 240 min due to our ELISA assay sensitivity. The absolute bioavailability (*F*) of bvPLA2 at each dose was also affected by the nonlinearity of AUC_0−t_, resulting in the observed differences between the 5 mg/kg dose group (60.4%) and other dose groups (15.5–20.4%). The bvPLA2 was not detectable in the urine over 24 h (Ae_24 h_) after SC administration of NCBV.

Recently, therapeutic efficacy of bvPLA2 was observed in the low-dose group (0.2 mg/kg) in mice [12]. Therefore, the nonlinearity of the AUC_0−t_ and *C*_max_ at the highest dose (5 mg/kg) observed herein would not be clinically meaningful. Some peptide drugs have been reported to exhibit nonlinear pharmacokinetics [18], and a target-mediated drug disposition model has been introduced to characterize this property [19]. To understand the nonlinear pharmacokinetics of bvPLA2, the target proteins and clearance mechanisms should be investigated.

### 2.5. Multiple Subcutaneous Administration of NCBV in Rats at a Dose of 0.5 mg/kg Once a Week for Three Weeks 

**Figure 6:**
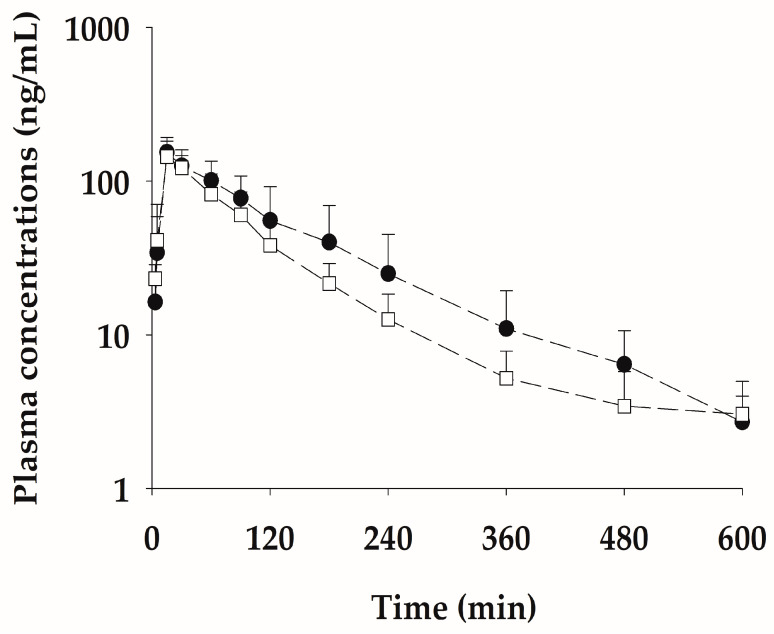
Mean plasma concentration-time profiles for bvPLA2 after single (□) and multiple (●) subcutaneous administration of NCBV at a dose of 0.5 mg/kg once a week for three weeks. Vertical bars represent SD.

Based on the improvement effect after administration once a week in Alzheimer’s disease mouse experiments [12,13], multiple dose studies with NCBV were conducted once a week for a total of three weeks. The pharmacokinetic profile of bvPLA2 after multiple SC administrations of NCBV is shown in Figure 6, and the pharmacokinetic parameters are listed in Table 5. No significant differences (*p* > 0.05) were observed in any of the bvPLA2 pharmacokinetic parameters between the multiple SC administration groups. The *C*_max_ of bvPLA2 in multiple and single doses was 154 ± 38.8 ng/mL and 145 ± 37.6 ng/mL, respectively, while AUC_0−t_ values were 19.0 ± 9.13 µg∙min/mL and 14.2 ± 4.46 µg∙min/mL, respectively. Excreted bvPLA2 was not detectable in the urine over 24 h in the NCBV group. The results suggested that multiple doses of NCBV (0.5 mg/kg/week) did not display different distribution and elimination features compared to single doses (0.5 mg/kg) and that bvPLA2 did not accumulate after multiple administrations. Additionally, no local tissue irritation, which could affect the rate of subsequent absorption of the drug, was found when administering the drug in the same area. 

### 2.6. Pharmacokinetic Properties of bvPLA2 after Subcutaneous Administration of the Pure Form Equivalent to 0.5 mg/kg NCBV

**Figure 7:**
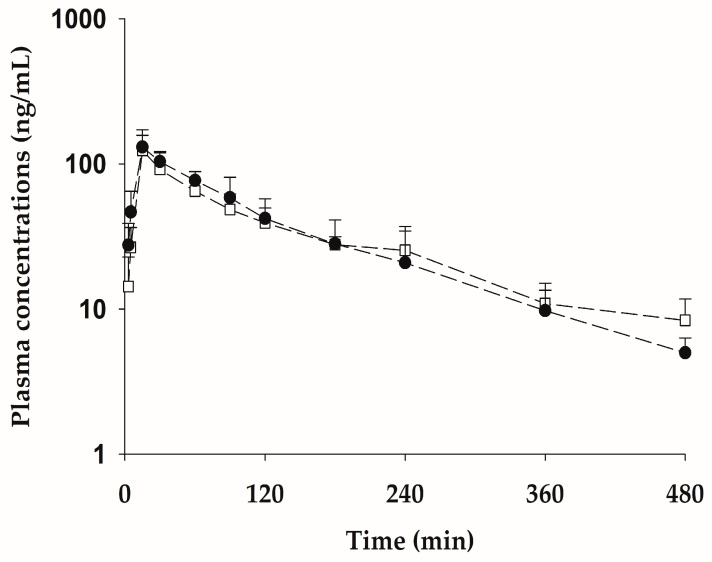
Mean plasma concentration-time profiles for bvPLA2 after subcutaneous administration of the pure form equivalent to 0.5 mg/kg of NCBV (bvPLA2 of NCBV: 0.5 mg/kg: □, bvPLA2 of pure form 0.381 mg/kg: ●). Vertical bars represent SD.

The mean plasma concentration-time curves after SC administration of the pure form of bvPLA2 (0.381 mg/kg), which is equivalent to the administration of 0.5 mg/kg of NCBV, are shown in Figure 7, and the relevant pharmacokinetic parameters are listed in Table 6. The AUC_0−t_ values of bvPLA2 after administration of NCBV and pure form were 14.0 ± 1.04 and 14.8 ± 4.22 μg·min/mL, t_1/2_ values were 146 ± 53.4 and 114 ± 34.9 min, *C*_max_ values were 124 ± 47.4 and 134 ± 25.8, and *T*_max_ values were 15 (15–15) and 15 (15–30), respectively (*p* > 0.05). These results indicate that no statistically significant differences were observed in the pharmacokinetic parameters of bvPLA2. Therefore, these results indicate that the presence of other compounds, such as melittin, in NCBV did not affect the pharmacokinetic properties of bvPLA2.

At first glance, pure bvPLA2 could be considered a better drug because unpredictable adverse drug reactions to other compounds in NCBV can be avoided. However, pure bvPLA2 is not cost-effective because of the high cost of extraction and the low purity (30–40%).

### 2.7. Tissue Distribution after Subcutaneous Administration of NCBV in Rats at a Dose of 0.5 mg/kg 

**Figure 8:**
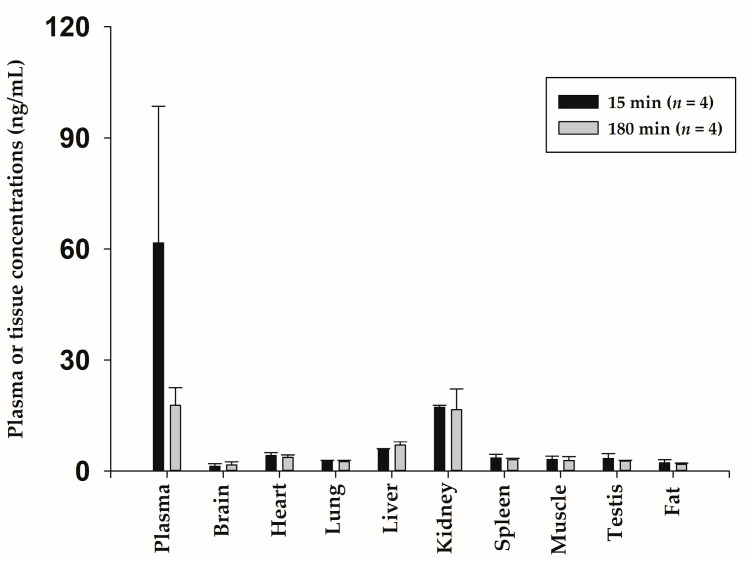
Mean concentration for bvPLA2 in various tissues at 15 min (*n* = 4) and 180 min (*n* = 4) after subcutaneous administration. Vertical bars represent SD.

Figure 8 shows the quantitative distribution of bvPLA2 in tissues (brain, heart, lung, liver, kidney, spleen, muscle, testis, and fat) at 15 min (*n* = 4) and 180 min (*n* = 4) after SC administration of NCBV at a dose of 0.5 mg/kg. At 15 min and 180 min, although it was detected in the brain, heart, lung, liver, kidney, spleen, muscle, testis, and fat homogenates, the corresponding bvPLA2 concentrations were lower than those in the plasma, respectively (Figure 8). The calculated tissue-to-plasma ratio for nine tissues was all <1.0, within the range of 0.0185 ± 0.00101 to 0.327 ± 0.147 at 15 min and 0.0856 ± 0.0146 to 0.994 ± 0.149 at 180 min. These data indicated that the rat organs studied had a low affinity for bvPLA2, which was supported by the considerably small V_dss_ value of IV-injected bvPLA2 (49.3 ± 1.79 mL/kg, Table 2), although a different route of administration was applied. It is well known that the V_dss_ of therapeutic peptides is usually small and does not undergo extensive tissue distribution [17]. Among the tested nine tissues, the highest tissue-to-plasma ratio at each time was observed in the kidney (0.327 ± 0.147 at 15 min, 0.994 ± 0.149 at 180 min, respectively), which plays a role in metabolism and excretion of proteins and peptides [17,20]. Although bvPLA2 showed high affinity for the kidney compared to other tissues, intact bvPLA2 was not detected in urine in all experimental groups. This means bvPLA2 was highly metabolized by proteolysis in the kidney and then excreted as metabolites. In this study, we did not identify the metabolites of bvPLA2, so further study is required to evaluate the metabolic profiling of bvPLA2.

## 3. Materials and Methods

### 3.1. Materials and Reagents

A new composition of bee venom (NCBV, lot no. NCBV014, containing 76.2% bvPLA2) was obtained from INISTst (Yongin, Gyeonggi-do, Republic of Korea). Bee venom phospholipase A2 (bvPLA2) was acquired from INISTst (Yongin, Gyeonggi-do, Republic of Korea). 

An ELISA kit for quantification of bvPLA2 in biological samples was obtained from Abclon (Seoul, Republic of Korea). The ELISA kit was based on a sandwich-type format. Coating: anti-bvPLA2 (#7G8) in carbonate buffer; Blocking Buffer: 3% skim milk in phosphate buffered saline (PBS); Standard: bvPLA2 in AbClon diluent 1; Washing Buffer: Tris-buffered saline, 0.1% Tween 20 (0.1% TBST); Secondary Antibody: Avidin-HRP, Substrate Solution (TMB); Detector Antibody: Anti-bvPLA2-biotin (#5E8); Stop Solution: 1N H_2_SO_4_. Benchtop Incubator: Thermo Scientific, model 311; ELISA plates: 96-well microplates; ELISA reader: SpectraMax 190 Microplate Reader (Molecular Devices, San Jose, CA, USA).

### 3.2. Calibration Standards and Quality Controls

Stock solutions of bvPLA2 (1 mg/mL) were prepared in PBS and serially diluted with standard dilution buffer. Concentrations of standard calibrations were 100, 50.0, 25.0, 12.5, 6.25, 3.13, 1.56, 0.78, and 0 ng/mL, adding one anchor point (0.39 ng/mL), and the concentrations of quality controls (QC) were 80, 10, and 2 ng/mL, respectively.

### 3.3. Sandwich ELISA Procedure and Assay Validation

An ELISA assay was performed using a bvPLA2 ELISA Kit (Abclon, Seoul, Republic of Korea). Briefly, a 96-well microplate was coated with 100 μL/well of a 1 μg/mL solution of anti-bvPLA2 (#7G8) in carbonate buffer and incubated at 4 °C overnight. The coated plate was kept at room temperature (25 °C) for 30 min before use. Each well was washed by adding 300 μL of washing buffer at room temperature for 5 min, and the remaining washing buffer was discarded. After washing the plates, 100 μL (of diluted standards, QCs, and samples) per well of 1:100 diluted calibrators, QCs, and samples were added in duplicate. The plates were incubated at 37 °C for 1 h and washed three times. One hundred microliters per well of detector antibody were added and incubated at 37 °C for 1 h. After washing the plate three times, 100 μL of secondary antibody was added to each well, and the plates were incubated for 30 min at room temperature. After washing thrice, 100 μL of substrate solution was added and stored for 10 min at room temperature. In each well, 100 μL of stop solution was added to stop the reaction. The absorbance was measured at 450 nm using a Spectramax 190 ELISA plate reader. The concentrations of bvPLA2 were interpolated using a 4-parameter logistic calibration curve implemented in SoftMax Pro 6.x software with a weighting factor of 1/C2.

The minimum required dilution was established by testing unspiked plasma diluted at 1:2, 1:4, 1:10, and 1:20 using a standard dilution buffer. Plasma samples were spiked with bvPLA2 to a final concentration of 25 ng/mL (*n* = 5 for each dilution).

The accuracy of the assay was assessed by low, medium, and high QCs, and relative errors (% RE = [measured concentration − nominal concentration]/nominal concentration) were calculated. Intra-day precision (CV%) was assessed on the same day and inter-day precision on six independent days. The nominal concentrations on the standard curve were defined when CV and RE were within 20%, except for the lowest and highest concentrations. The lowest and highest concentrations on the standard curve were defined as the lower limit of quantification (LLOQ) and upper limit of quantification (ULOQ) when CV and RE were within 25%, according to the US FDA bioanalytical method validation guidance for industry [16]. 

### 3.4. Animals

Male Sprague-Dawley rats (7–9 weeks, 230–330 g) were purchased from Young Bio (Seongnam, Gyeonggi-do, Republic of Korea). The animal study protocol was approved by the Department of Laboratory Animals, Institutional Animal Care, and Use Committee on the Songsim Campus of the Catholic University of Korea (Approval No. 2019-040). The rats were maintained under controlled environmental conditions (temperature 20 ± 2 °C; relative humidity 55 ± 5%; 12-h light/dark cycle).

### 3.5. Pharmacokinetic Studies of bvPLA2 after Intravenous Administration of NCBV in Rats

The jugular vein (for IV administration) and carotid artery (for blood sampling) of each rat were cannulated with a polyethylene tube. Rats received a single dose of 0.05 mg/kg NCBV dissolved in normal saline as an intravenous bolus (*n* = 5). Blood samples were collected via the carotid artery at 0 (pre-dose), 1, 3, 5, 15, 30, 60, 90, 120, 180, 240, 360, 480, 600, and 1440 min after the IV bolus. Then, 0.3 mL of a heparinized 0.9% NaCl-injectable solution (20 units/mL) was used to flush the cannula immediately after each blood sampling attempt to prevent blood clotting. The blood samples were immediately centrifuged, and an approximately 120 μL aliquot of each plasma sample was stored at −80 °C until further use in the ELISA experiment. At the end of 24 h, each metabolic cage was rinsed with 20 mL of distilled water, and the rinsings were combined with the 24 h urine sample. After determining the exact volume of the combined urine samples, they were stored at −80 °C until further use in the ELISA quantification. At the same time (24 h), each rat was euthanized using CO_2_.

### 3.6. Pharmacokinetic Studies of bvPLA2 after Subcutaneous Administration of NCBV in Rats 

Single-dose pharmacokinetic studies were conducted on 25 rats. The rats were randomly divided into five groups (*n* = 5 per group) and subcutaneously administered NCBV at doses of 0.2, 0.5, 1, 2, and 5 mg/kg to assess whether the pharmacokinetic profile of bvPLA2 followed a dose-dependent linear relationship. Blood samples were collected via the carotid artery at 0 (pre-dose), 3, 5, 15, 30, 60, 90, 120, 180, 240, 360, 480, 600, and 1440 min after SC administration of NCBV.

For the pharmacokinetic study of multiple administrations, rats were randomly divided into two groups (*n* = 5 per group). One group was subcutaneously administered 0.5 mg/kg of NCBV once a week for two weeks, and the other group was subcutaneously administered normal saline once a week for two weeks. In the third week, rats in each group were subcutaneously administered 0.5 mg/kg of NCBV. Blood samples were collected at 0, 3, 5, 15, 30, 60, 90, 120, 180, 240, 360, 480, 600, and 1440 min after the administration.

In the comparative study between NCBV and pure bvPLA2, rats were randomly divided into two groups (*n* = 5 per group). NCBV (Lot No. NCBV014) at a dose of 0.5 mg/kg and bvPLA2 standard at a dose of 0.381 mg/kg were subcutaneously administered to the animals. The purity and content of bvPLA2 in NCBV were determined to be 76.2% using HPLC (Figure 2B). The other procedures were the same as those used in the single intravenous administration study.

### 3.7. Tissue Distributions of bvPLA2 following Subcutaneous Administration of NCBV 

In the tissue distribution study, NCBV was dissolved in the same vehicle used in the SC studies and administered to the rats at 0.5 mg/kg. Blood from each rat (*n* = 4 from each group) was collected from the abdominal aorta after 15 and 180 min, and each rat was euthanized with CO_2_. After centrifugation of each blood sample, two 100 μL plasma aliquots were stored at −20 °C until the ELISA analysis. Following complete systemic perfusion with 0.9% injectable NaCl solution, approximately 1 g of brain, heart, lung, liver, kidney, spleen, muscle, testis, and fat samples were collected, washed with 0.9% injectable NaCl solution, and blotted dry with tissue paper. An accurately weighted amount of each tissue sample was homogenized in ELISA standard dilution buffer at 1:2 (*w/v*) using a tissue homogenizer. The mixture was centrifuged at 14,000 rpm for 10 min at 4 °C, and the supernatant was stored at −20 °C until ELISA. To reduce the matrix effects, the calibration curves were obtained by adding the standard substances to the samples. The concentration range of bvPLA2 in various tissues of rats was 0.78–100 ng/mL.

### 3.8. Pharmacokinetic and Statistical Analysis 

Pharmacokinetic parameters were determined by non-compartmental analysis using Phoenix WinNonlin^®^ (version 6.0; Certara USA, Princeton, NJ, USA) to calculate the total area under the plasma concentration-time curve from zero to time infinity (AUC_inf_) or to the last measured time (AUC_0−t_), the peak plasma concentration (*C*_max_), and time to reach *C*_max_ (*T*_max_), total body clearance (CL), mean residence time (MRT), apparent volume of distribution at steady state (V_dss_), terminal half-life (t_1/2_), and bioavailability (*F*) [21]. The SC bioavailability (*F*) of bvPLA2 was calculated as follows:

*F* = (AUC_sc_/ACU_iv_) × dose_iv_/dose_sc_) × 100 (1)

where AUC_sc_ and AUC_iv_ are the AUC_inf_ values after the SC and IV administrations of NCBV, respectively. 

All pharmacokinetic data were summarized using descriptive statistics. All results are expressed as the mean ± standard deviation (SD), except for *T*_max_, which was described as the median (range). Statistical analyses were performed using the Statistical Package for the Social Sciences (SPSS, version 25; IBM Corp., Armonk, NY, USA). The statistical significance was determined at *p* < 0.05 using a *t*-test between the two means for unpaired data. A power model (Y = α*(dose)^β^) modified by Smith et al. [22] was used to assess the dose proportionality of the pharmacokinetic parameters (AUC_0−t_ and *C*_max_). Y represents the parameters (AUC_0−t_ and *C*_max_), α is the scale parameter, and β is the proportionality exponent to be estimated. Dose proportionality was declared when the 90% confidence interval (CI) of slope β lay within the acceptance range [1 + log(Θ_L_)/log(r), 1 + log(Θ_H_)/log(r)], where Θ_L_ is 0.8 as the low limit and Θ_H_ is 1.25 as the high limit of the CI and *r* is 25, corresponding to the dose ratio between the highest dose (5 mg/kg) and the lowest dose (0.2 mg/kg). 

## 4. Conclusions

In this study, we have prepared and used the NCBV (a fortified content up to 76.2% bvPLA2), which consisted of complex mixtures such as peptides, phospholipids, bioactive amines, sugars, enzymes, and minerals, to develop a new drug for use in patients with Alzheimer′s disease. 

Since PLA2 was extracted and purified from cobra venom in early 1980, PLA2 has been discovered in various organisms such as bacteria, fungi, plants, scorpions, snakes, and bees [23]. These PLA2s have a wide range of pharmacological properties, such as anti-bacterial, anti-cancer, anti-viral, anti-inflammatory, anti-nociceptive, and defense against neurodegenerative diseases [2,3,4,7,23,24,25]. Of these, especially bvPLA2, which belongs to group III secretory PLA2 with a molecular weight of 14 to 18 kDa and is known to be 31% identical to mammalian group III sPLA2 [7]. It has been demonstrated that bvPLA2 has a therapeutic effect on Alzheimer’s disease, with targets described in Figure 1 [8,9,10,11,12,13]. Based on this research, we paid attention to the pharmacological effect of bvPLA2 and evaluated the pharmacokinetic properties of NCBV for safe clinical usage.

To the best of our knowledge, there is currently no information available on the pharmacokinetics of bvPLA2 in rats. Therefore, we characterized the pharmacokinetic properties of bvPLA2 after IV and SC administrations of NCBV in rats. bvPLA2 exhibited nonlinear pharmacokinetics at various doses after SC administration; thus, further studies on bvPLA2 distribution and clearance are required. In the multiple-dose study, no differences were observed between the multiple-dose and single-dose groups. Furthermore, other constituents of NCBV did not influence the pharmacokinetic profile of bvPLA2 when compared to pure bvPLA2. In the additional tissue distribution study, the tissue-to-plasma ratio for nine tissues was all <1.0 both at 15 min and 180 min, representing a limited distribution of the bvPLA2 within the tissues. The findings of this study may help understand the pharmacokinetic characteristics of bvPLA2 and provide useful information for further clinical studies on NCBV.

Alzheimer′s dementia is a disease with an increasing incidence worldwide, according to an Alzheimer′s association study. As of 2022, approximately 6.5 million Americans over the age of 65 have Alzheimer′s dementia, and by 2050, the number of people aged 65 and older with Alzheimer′s dementia is projected to reach 12.7 million [26]. Treatments for neurodegenerative diseases currently performed in clinical settings aim to slow disease progression or alleviate cognitive function and behavioral symptoms. 

The pathogenesis of Alzheimer′s dementia is highly diverse and still under investigation, leading to the development of treatments with different mechanisms of action. In addition to bvPLA2 (group III sPLA2), lipoprotein-associated PLA2 (Lp-PLA2) has been associated with inflammatory markers and Alzheimer’s disease, and strategies to treat Alzheimer’s disease have been proposed [27]. Clinical trials are currently underway for the Lp-PLA2 inhibitors rilapladib and GSK2647544, which are being developed for the treatment of Alzheimer’s disease (**ClinicalTrials.gov** Identifier: NCT01428453 and NCT01702467). Like Lp-PLA2 inhibitors, NCBV might be a novel therapeutic for Alzheimer’s disease because its supposed mechanism is the inhibition of nerve cell destruction, indicating a protective effect rather than just alleviating the symptoms. 

## Figures and Tables

**Table 1:** Minimum required dilution of bvPLA2 for measurement of plasma bvPLA2 levels.

Dilution Factor	Theoretical (ng/mL)	Measured (ng/mL)	% of the Theoretical Value Accuracy (Measuresd/Theoretical × 100)	Standard Deviation (SD)
1:2	25	24.1	96.4	0.853
1:4	25	25.4	102	1.36
1:10	25	25.8	103	0.741
1:20	25	26.6	106	2.32

**Table 2:** Pharmacokinetic parameters (mean ± SD) of bvPLA2 after a single intravenous administration of NCBV in rats at a dose of 0.05 mg/kg.

PK Parameters	0.05 mg/kg (*n* = 5)
AUC_0−t_ (µg∙min/mL) ^1^	11.8 ± 0.869
AUC_0−inf_ (µg∙min/mL) ^2^	11.8 ± 0.866
t_1/2_ (min) ^3^	8.10 ± 0.669
V_dss_ (mL/kg) ^4^	49.3 ± 1.79
MRT (min) ^5^	8.11 ± 1.07
CL (mL/min/kg) ^6^	4.24 ± 0.291
Ae_24 h_ (% of dose) ^7^	ND ^8^

^1^ Total area under the plasma concentration-time curve from time zero to time last sampling time. ^2^ Total area under the plasma concentration-time curve from time zero to infinity. ^3^ Terminal half-life. ^4^ Apparent volume of distribution at steady state. ^5^ Mean residence time. ^6^ Total body clearance. ^7^ Percentages of bvPLA2 excreted over the 24-h urine. ^8^ Not detected.

**Table 3:** Pharmacokinetic parameters (mean ± SD) of bvPLA2 in rats after single subcutaneous administration of NCBV at doses of 0.2, 0.5, 1, 2, and 5 mg/kg.

PK Parameters	0.2 mg/kg (*n* = 5)	0.5 mg/kg (*n* = 5)	1 mg/kg (*n* = 5)	2 mg/kg (*n* = 5)	5 mg/kg (*n* = 5)
Body weight (g)	309 ± 10.9	314 ± 11.3	350 ± 14.4	267 ± 3.88	265 ± 9.84
AUC_0−t_ (µg∙min/mL) ^1^	9.16 ± 1.66	19.0 ± 5.09	43.8 ± 6.71	64.9 ± 8.61	645 ± 9.55
AUC_0-inf_ (µg∙min/mL) ^2^	9.68 ± 2.00	20.3 ± 6.46	45.4 ± 7.82	73.4 ± 10.2	715 ± 3.58
t_1/2_ (min) ^3^	63.1 ± 8.12	150 ± 111	197 ± 88.3	169 ± 39.5	170 ± 14.6
*C*_max_ (ng/mL) ^4^	91.9 ± 11.2	161 ± 28.3	287 ± 26.3	498 ± 51.2	2480 ± 195
*T*_max_ (min) ^5^	22.5 (15–30)	15 (15–90)	15 (15–15)	15 (15–60)	60 (30–90)
Ae_24 h_ (% of dose) ^6^	ND ^7^	ND	ND	ND	ND
V_dss_/*F* (L/kg) ^8^	1.93 ± 0.376	4.49 ± 2.37	5.23 ± 3.31	6.95 ± 1.24	1.34 ± 0.111
*F* (%) ^9^	20.4	17.1	19.2	15.5	60.4

^1^ Total area under the plasma concentration-time curve from time zero to time last sampling time. ^2^ Total area under the plasma concentration-time curve from time zero to infinity. ^3^ Terminal half-life. ^4^ Peak plasma concentration. ^5^ Time to reach *C*_max_: Median (ranges). ^6^ Percentages of bvPLA2 excreted over the 24-h urine. ^7^ Not detected. ^8^ Apparent volume of distribution at steady state/bioavailability. ^9^ Subcutaneous bioavailability.

**Table 4:** Assessment of dose proportionality of bvPLA2 on the power model.

Parameter	Linearity Range (mg/kg)	R^2^	Dose Proportionality Coefficient (β)	90% CI ^3^	Acceptance Range
*C*_max_^1^	0.2–5	0.914	1.01 ± 0.0774	0.873–1.14	0.93–1.06
AUC_0−t_^2^	0.2–5	0.907	1.28 ± 0.102	1.11–1.46	0.93–1.06

^1^ Peak plasma concentration. ^2^ Total area under the plasma concentration-time curve from time zero to time last sampling time. ^3^ 90% confidence interval.

**Table 5:** Pharmacokinetic parameters (mean ± SD) of bvPLA2 in rats after multiple and single subcutaneous administrations of NCBV at doses of 0.5 mg/kg.

PK Parameters	Multiple (*n* = 5)	Single (*n* = 5)
AUC_0−t_ (µg∙min/mL) ^1^	19.0 ± 9.13	14.2 ± 4.46
t_1/2_ (min) ^2^	95.4 ± 18.0	110 ± 44.2
*C*_max_ (ng/mL) ^3^	154 ± 38.8	145 ± 37.6
*T*_max_ (min) ^4^	15 (15–15)	15 (15–15)
Ae_24 h_ (% of dose) ^5^	ND ^6^	ND

^1^ Total area under the plasma concentration-time curve from time zero to time last sampling time. ^2^ Terminal half-life. ^3^ Peak plasma concentration. ^4^ Time to reach *C*_max_: Median (ranges). ^5^ Percentages of bvPLA2 excreted over the 24-h urine. ^6^ Not detected.

**Table 6:** Pharmacokinetic parameters (mean ± SD) of bvPLA2 in rats after single subcutaneous administration of NCBV at a dose of 0.5 mg/kg and bvPLA2 pure form (0.381 mg/kg).

PK Parameters	NCBV (*n* = 5)	bvPLA2 Pure form (*n* = 5)
AUC_0−t_ (µg∙min/mL) ^1^	14.0 ± 1.04	14.8 ± 4.22
t_1/2_ (min) ^2^	146 ± 53.4	114 ± 34.9
*C*_max_ (ng/mL) ^3^	124 ± 47.4	134 ± 25.8
*T*_max_ (min) ^4^	15 (15–15)	15 (15–30)
Ae_24 h_ (% of dose) ^5^	ND ^6^	ND

^1^ Total area under the plasma concentration-time curve from time zero to time last sampling time. ^2^ Terminal half-life. ^3^ Peak plasma concentration. ^4^ Time to reach *C*_max_: Median (ranges). ^5^ Percentages of bvPLA2 excreted over the 24-h urine. ^6^ Not detected.

## Data Availability

Not applicable.
